# Use of anti-amyloid therapies for Alzheimer’s disease in Brazil: a position paper from the Scientific Department of Cognitive Neurology and Aging of the Brazilian Academy of Neurology

**DOI:** 10.1590/1980-5764-DN-2024-C002

**Published:** 2024-11-11

**Authors:** Breno José Alencar Pires Barbosa, Elisa de Paula França Resende, Raphael Machado Castilhos, Wyllians Vendramini Borelli, Norberto Anízio Ferreira Frota, Marcio Luiz Figueredo Balthazar, Augusto Celso Scarparo Amato, Jerusa Smid, Maira Tonidandel Barbosa, Artur Martins Coutinho, Leonardo Cruz de Souza, Lucas Porcello Schilling, Mari Nilva Maia da Silva, Gustavo Bruniera Peres Fernandes, Paulo Henrique Ferreira Bertolucci, Ricardo Nitrini, Eliasz Engelhardt, Orestes Vicente Forlenza, Paulo Caramelli, Sonia Maria Dozzi Brucki, Adalberto Studart-Neto

**Affiliations:** 1 Academia Brasileira de Neurologia, Departamento Científico de Neurologia Cognitiva e do Envelhecimento, São Paulo SP, Brazil.; 2 Universidade Federal de Pernambuco, Centro de Ciências Médicas, Área Acadêmica de Neuropsiquiatria, Recife PE, Brazil.; 3 Universidade Federal de Pernambuco, Hospital das Clínicas, Empresa Brasileira de Serviços Hospitalares, Serviço de Neurologia, Recife PE, Brazil.; 4 Universidade Federal de Minas Gerais, Faculdade de Medicina, Unidade de Neurologia Cognitiva e do Comportamento, Belo Horizonte MG, Brazil.; 5 Hospital de Clínicas de Porto Alegre, Serviço de Neurologia, Centro de Neurologia Cognitiva e Comportamental, Porto Alegre RS, Brazil.; 6 Universidade Federal do Rio Grande do Sul, Instituto de Ciências Básicas da Saúde, Departamento de Ciências Morfológicas, Porto Alegre RS, Brazil.; 7 Hospital Geral de Fortaleza, Serviço de Neurologia, Fortaleza CE, Brazil.; 8 Universidade de Fortaleza, Fortaleza CE, Brazil.; 9 Universidade Estadual de Campinas, Faculdade de Ciências Médicas, Departamento de Neurologia, Campinas SP, Brazil.; 10 Universidade Estadual de Campinas, Faculdade de Ciências Médicas, Departamento de Radiologia, Campinas SP, Brazil.; 11 Universidade de São Paulo, Faculdade de Medicina, Hospital das Clínicas, Departamento de Neurologia, Grupo de Neurologia Cognitiva e do Comportamento, São Paulo SP, Brazil.; 12 Universidade de São Paulo, Faculdade de Medicina, Hospital das Clínicas, Instituto de Radiologia, Centro de Medicina Nuclear, Laboratório de Investigação Médica (LIM 43), São Paulo SP, Brazil.; 13 Hospital Sírio-Libanês, Medicina Nuclear e Serviço de PET-CT, São Paulo SP, Brazil.; 14 Pontifícia Universidade do Rio Grande do Sul, Escola de Medicina, Serviço de Neurologia, Porto Alegre RS, Brazil.; 15 Hospital Nina Rodrigues, Serviço de Neuropsiquiatria, São Luís MA, Brazil.; 16 Hospital Israelita Albert Einstein, Laboratório Clínico, São Paulo SP, Brazil.; 17 Universidade Federal de São Paulo, Escola Paulista de Medicina, Departamento de Neurologia e Neurocirurgia, São Paulo SP, Brazil.; 18 Universidade Federal do Rio de Janeiro, Instituto de Neurologia Deolindo Couto, Rio de Janeiro RJ, Brazil.; 19 Universidade Federal do Rio de Janeiro, Instituto de Psiquiatria, Rio de Janeiro RJ, Brazil.; 20 Universidade de São Paulo, Faculdade de Medicina, Hospital das Clínicas, Instituto de Psiquiatria, Laboratório de Neurociências, São Paulo SP, Brazil.

**Keywords:** Alzheimer Disease, Amyloid, Therapeutics, Antibodies, Monoclonal, Doença de Alzheimer, Amiloide, Terapêutica, Anticorpos Monoclonais

## Abstract

Novel therapies for Alzheimer’s disease, particularly anti-amyloid drugs like lecanemab and donanemab, have shown modest clinical benefits but also significant risks. The present paper highlights the challenges of access to diagnosis, cost-effectiveness, safety, and the need for more representation of diverse populations in clinical trials. Recommendations include careful patient selection, risk-benefit analysis, and the importance of proven amyloid pathology for treatment. Future work involves further research on anti-amyloid therapies in Brazil and the development of more effective treatments for Alzheimer’s disease.

## RESUMO

Novas terapias para a doença de Alzheimer, particularmente os anticorpos monoclonais antiamiloides como o lecanemab e o donanemab, demonstraram benefícios clínicos modestos, mas também riscos significativos. O presente artigo destaca os desafios do acesso ao diagnóstico, da relação custo-eficácia, da segurança e da necessidade de maior representação de populações diversas em ensaios clínicos. As recomendações incluem seleção cuidadosa dos pacientes, análise de risco-benefício e a importância da patologia amiloide comprovada para o tratamento. Trabalhos futuros envolvem mais pesquisas sobre terapias antiamiloides no Brasil e o desenvolvimento de tratamentos mais eficazes para a doença de Alzheimer.

## INTRODUCTION

Novel therapies that seek to tackle the pathophysiology of Alzheimer’s disease (AD) have been tested in randomized clinical trials, and some of them have gained attention due to statistically significant differences in clinical outcomes in comparison to placebo^1^. The first group of drugs to enter clinical practice in recent years are monoclonal antibodies directed to the amyloid-β protein (Aβ), which bind to different species of the protein aggregation chain. This treatment approach is based on the “amyloid cascade hypothesis”, which states that Aβ aggregation triggers a cascade of pathophysiological events, including synaptic and network dysfunction, neuroinflammation, aggregation, and spreading of phosphorylated tau (p-tau) tangles. The propagation of p-tau is associated with synaptic loss and neurodegeneration, culminating in cognitive decline and dementia. By binding to Aβ aggregates, monoclonal antibodies facilitate the clearance of Aβ from the brain, potentially reducing both direct and downstream deleterious effects of Aβ and, therefore, delaying cognitive and functional decline^2^.

Since the controversial approval of aducanumab in July 2021 by the United States Food and Drugs Administration (FDA), new clinical trials have published heterogeneous results from different anti-amyloid agents. The FDA approval of aducanumab was based on a surrogate endpoint deemed “reasonably likely to predict clinical benefit”, given its property to target and clear amyloid aggregates from the brain. However, the drug was not approved by other agencies, such as the European Medicines Agency (EMA) and the Brazilian Health Regulatory System (ANVISA, *Agência Nacional de Vigilância Sanitária*), under the justification that there was a lack of evidence that clinical benefits outweigh the risks of the treatment. During the subsequent two years, challenges related to the clinical use of aducanumab, including controversies over its effectiveness and high price, led to discontinuing the development and commercialization of aducanumab in January 2024^3^.

Lecanemab, a monoclonal antibody primarily targeting soluble amyloid protofibrils, was the second anti-amyloid drug to receive accelerated approval by the FDA in January 2023. In July of the same year, accelerated approval was converted to definitive based on phase 3 CLARITY-AD trial data. This study, which enrolled 1,795 participants, showed a relative 27% slowing of clinical decline on the Clinical Dementia Rating — Sum of Boxes (CDR-SB) scale compared to placebo in people with mild cognitive impairment (MCI) or mild dementia due to AD during 18-month follow-up^4^. Noteworthy, this relative difference is equivalent to an absolute 0.45 points difference in the same score, which ranges from 0 to 18. In July 2024, the EMA refused marketing authorization for lecanemab in the European Union. The EMA’s human medicines committee considered that the benefits of treatment are not significant enough to outweigh the risks of serious adverse events associated with lecanemab^5^.

A third anti-amyloid drug, donanemab, binds to the N-terminal truncated form of Aβ and aids plaque removal through microglial-mediated phagocytosis. The TRAILBLAZER-ALZ 2 randomized clinical trial was a placebo-controlled, 18-month-long phase 3 trial that enrolled 1,736 participants with early symptomatic AD to assess the efficacy and adverse events of donanemab^6^. In the TRAILBLAZER-ALZ 2 trial, the group receiving donanemab experienced a statistically significant 35% reduction in clinical decline compared to placebo using a scale that assesses cognition and activities of daily living (iADRS, Integrated Alzheimer’s Disease Rating Scale) and 29% slowing on the CDR-SB. However, this relative difference in absolute numbers corresponded to only 3.25 points on the 144-point iADRS scale^6^. In July 2024, FDA approved donanemab for the treatment of early symptomatic AD.

While the modest slowing of cognitive and functional decline demonstrated by these drugs was welcomed as a positive starting point, debates regarding the limitations of the novel AD treatments with monoclonal antibodies targeting Aβ have gained increasing interest from society. Issues such as access to diagnosis using biomarkers, eligibility in a “real-life scenario,” clinical meaningfulness, cost-effectiveness, safety and adverse events, inequalities, and financing remain significant challenges to be debated, especially in low- and middle-income countries (LMIC), such as Brazil^7^.

Aligned with international medical societies and concerned with the challenging scenario presented in Brazil for the incorporation of new anti-amyloid therapies for AD, the Scientific Department of Aging and Cognitive Neurology of the Brazilian Academy of Neurology appointed a multidisciplinary group of experts to form a task force dedicated to the topic. This manuscript aims to critically discuss:

- The modest clinically meaningful effect size of such treatments and eligibility in a “real-life scenario”;
- Recommendations for appropriate use of anti-amyloid therapies in Brazil;
- High costs that may significantly burden the Brazilian public and private health systems; and
- The risk of indiscriminate use for non-eligible patients (e.g., asymptomatic individuals or moderate-to-advanced stages of dementia).

This manuscript is not intended to endorse the approval of these medications by regulatory agencies but rather to discuss the impact of anti-amyloid therapies from the perspective of individual patient care and public health. Therefore, the group seeks to raise necessary topics for discussion and critically address paths in the field.

## METHODS

The present position paper was prepared by specialists and researchers in dementia and AD (Neurologists, Geriatricians, Psychiatrists, Nuclear Medicine Physicians, Neuroradiologists, and Clinical Pathologists). A focused literature review was carried out based on searches in the MEDLINE, Scopus, SciELO, and LILACS databases until July 2024, using the descriptors “Alzheimer’s disease” AND “monoclonal antibodies”. We mainly selected articles published in the last ten years, although older relevant publications were not discarded. Only articles in English were reviewed. The authors met several times for debate, and the points of consensus constituted the recommendations.

### Assessment of amyloid status

Proven amyloid pathology is pivotal to prescribing anti-amyloid drugs. Although not necessary for anti-amyloid therapies’ eligibility, confirmation of tau pathology may also provide vital information for individuals with clinical indications for anti-amyloid therapy. Amyloid pathology can be identified by both positron emission tomography (PET) or lumbar puncture with cerebrospinal fluid (CSF) analyses (Table 1)^8,9,10,11^.

**Table 1:** Recommended biomarkers to identify the amyloid status available in Brazil.

PET markers	Cerebrospinal fluid markers
[^11^C]-PIB	Aβ42:Aβ40 ratio
[^18^F]-Florbetaben	p-tau181/Aβ42 ratio

Abbreviations: PET, positron emission tomography; Aβ, amyloid beta; p-tau, phosphorylated tau protein.

Regarding amyloid positivity, PET studies are generally the gold standard for detecting amyloid plaques *in vivo*. The diagnostic accuracy of clinically available amyloid tracers is similar and provides adequate sensitivity and specificity to identify amyloid plaques in the brain. International consensus recommends that PET positivity or negativity for significant Aβ deposition must be based on a visual qualitative reading by a board-certified nuclear medicine physician specialized in PET who has completed the formal training provided by the tracer manufacturer^12^. In cases where the results are indeterminate or unclear, we recommend performing a consensus reading by two physicians or quantitative measures like the centiloid scale or standardized uptake value ratio (SUVr). This approach has reduced the number of scans rated as indeterminate and raised the interrater agreement among experienced physicians in our reality^13^. The reference region for normalization in the SUVr/centiloid scale calculus is usually the mean whole cerebellum uptake^14,15^.

Amyloid levels are also measurable in the CSF. The ratios of Aβ42/Aβ40 and p-tau181/Aβ42 show the best correlations with amyloid-PET than Aβ42 alone^11^. Caution should be exercised in interpreting CSF biomarkers, which are typically associated with this method’s pre-analytical and analytical aspects^16^. When the physician requests CSF biomarkers, they must ensure that the laboratory follows the quality control recommendations of the Alzheimer’s Association and which analytical method is used (for example, automated methods are recommended)^17^.

Blood-based biomarkers (BBM) have demonstrated increasing diagnostic properties related to the AD pathological process. However, according to the most recent Alzheimer’s Association guidelines, BBM should be carefully indicated in symptomatic individuals as a supporting diagnostic tool or as a screening for other confirmatory and expensive biomarkers but not as a standalone exam^18^. Some caveats should be discussed thoroughly before implementing BBM in clinical practice, such as the influence of chronic kidney disease, its profile in diverse populations (thus prompting the need for local normative studies), and other confounding factors that might influence the interpretation of BBM. Their usage in clinical practice is promising but has yet to be recommended. Among plasma biomarkers, the most promising is p-tau217. Studies show that p-tau217 has the strongest association with amyloid stratus measured by amyloid PET or CSF Aβ42/Aβ40^19,20^.

In addition to molecular biomarkers, many topographical or neurodegenerative markers were studied to evaluate the downstream pathological changes of AD. Examples include medial temporal lobe atrophy and reduced glucose metabolism in the temporoparietal region^16^. Although these methods may assist in predicting outcomes, they are not indicated as standalone methods to measure amyloid or tau pathology in AD^10^. They are not considered appropriate for estimating amyloid burden.

### Eligibility and minimum resources for the safe and effective use of anti-amyloid therapies

Although the main criterion for the prescription of anti-amyloid therapies is the presence of amyloid pathology detected through biomarkers (see previous item), not all individuals with underlying AD pathology will benefit from anti-amyloid therapies. The most fundamental principle is that potential candidates for anti-amyloid therapies are in the early, symptomatic stages of AD, namely MCI or mild dementia. Despite some variation in the proportion of each stage of the disease among the studies, the majority of individuals included had a CDR global score of 0.5 or 1.0 and a CDR-SB score varying from 2.4 to 4.0 points (Table 2)^4,6^. Therefore, we recommend that these medications should not be prescribed to cognitively unimpaired individuals (e.g., asymptomatic or those with subjective cognitive decline [SCD]) or patients with moderate or severe stages of dementia due to AD with a CDR global score of 2.0 or 3.0 (moderate and severe dementia, respectively).

**Table 2:** Proportions of each clinical stage, global, and Clinical Dementia Rating Scale – Sum of Boxes from the studies of anti-amyloid drugs that showed clinical benefit.

	Drug		Donanemab	Lecanemab
	Study name		TRAILBLAZER-ALZ 2	CLARITY-AD
Clinical stage (%)		MCI	17.1	61.5
		Mild dementia	82.9	38.5
Global CDR score (%)*		0.5	60.8	80.8
		1.0	36.0	19.2
CDR-SB (max. 18) (mean±sd)*			4.0±2.1	3.2±1.3

Abbreviations: MCI, mild cognitive impairment; CDR-SB, Clinical Dementia Rating – Sum of Boxes Scale; sd, standard deviation.

Note: *obtained from the active treatment group.

**Figure 1:**
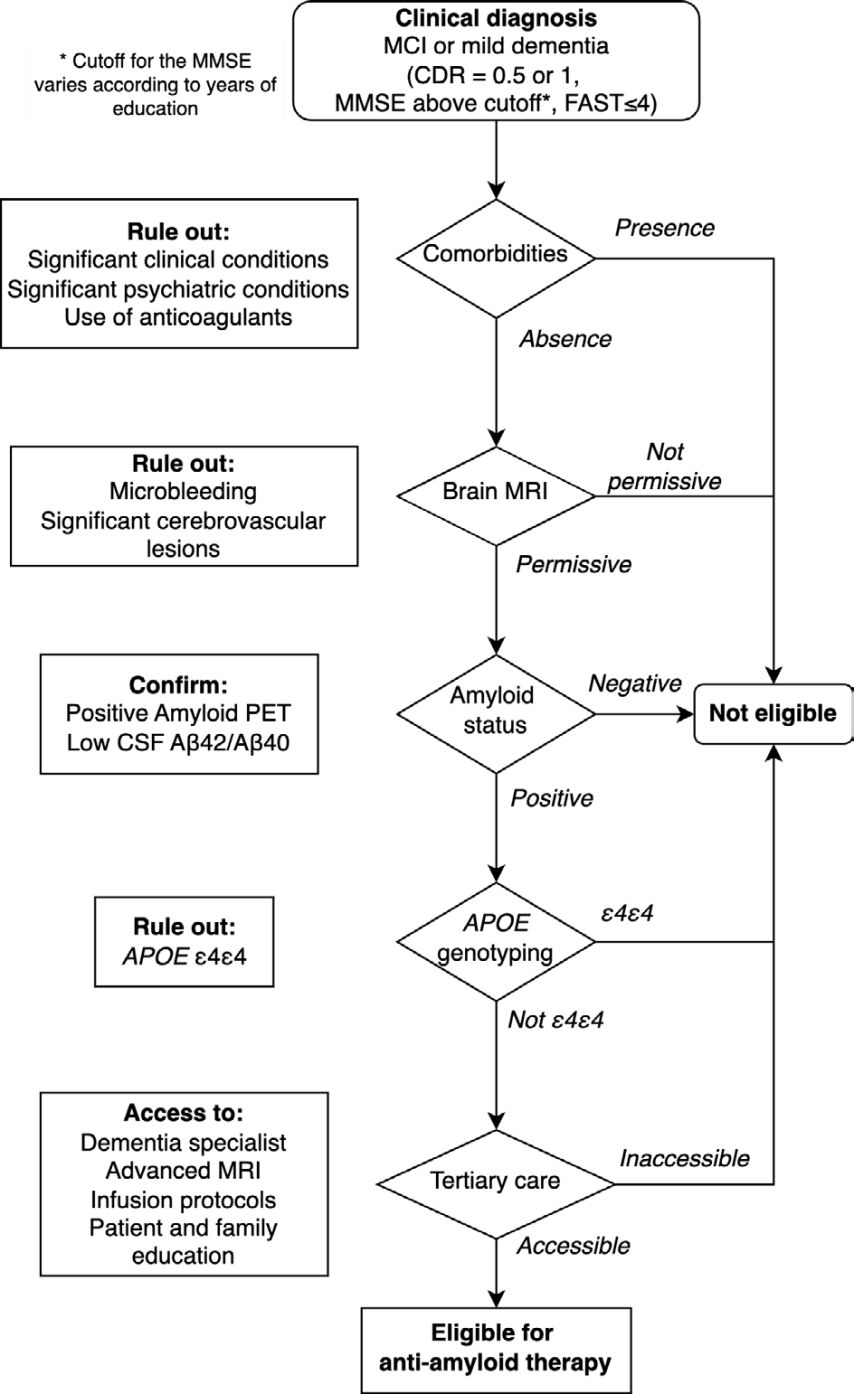
Flowchart for suggested eligibility criteria for anti-amyloid therapy.

Abbreviations: MCI, mild cognitive impairment; CDR, Clinical Dementia Rating; MMSE, Mini-Mental State Examination; FAST, Functional Assessment Staging Tool; MRI, magnetic resonance imaging; PET, positron emission tomography; Aβ, amyloid beta; CSF, cerebrospinal fluid; ApoE, apolipoprotein E gene.

We strongly suggest strictly following the inclusion and exclusion criteria of the trials for each drug (Table 3)^4,6^. In Boxes 1 and 2 ^8^, we propose indications and contraindications for the main anti-amyloid drugs, donanemab and lecanemab. In Box 3, we list the minimum resources the center should have to be considered safe to prescribe anti-amyloid therapies. Figure 1 shows a proposed flowchart for eligibility assessment.

**Table 3:** Summary of inclusion and exclusion criteria in the studies CLARITY-AD^4^ and TRAILBLAZER-ALZ 2^6^.

			Donanemab (TRAILBLAZER-ALZ 2)	Lecanemab (CLARITY-AD)
MCI or mild dementia	MMSE in the sample		20–28	≥22
	CDR		-	Global 0.5 or 1.0 | Memory ≥0.5
	Other		gradual, progressive memory change for ≥6 months	gradual decline in the past year, corroborated
	AD biomarkers	Amyloid PET	positive scan and	positive scan or
		CSF	-	t-tau/ Aβ^42^
		Tau PET	positive scan	-
Characteristics	Age		60–85	50–90
	Sex (% female)		53.3	52.0
	Race		White 94.9%	White >75.0%
			Black 2.9%	Black ~2.5%
			Asian 1.1%	Asian 17.0%
	BMI		-	17–35
	AChE-i or memantine		if stable for 30+ days	if stable for 12+ weeks
	Care partner		Yes	yes
Excluded health conditions	cause of cognitive impairment		any non-AD condition	any non-AD condition
	Laboratory tests		elevated liver enzymes	low B12, high TSH
	TIA/stroke		-	past year
	Seizure		recurrent seizures (except childhood febrile seizures)	past year
	Immune/Allergy		significant multiple or severe drug allergies	immunologic disease that is uncontrolled or requires systemic therapy
	Psychiatric		interfering, actively suicidal, or chronic psychosis	interfering, GDS ≥8, suicidal behavior (5 years)
	Cancer		past five years (except those with a low risk of spread)	past three years (except skin/prostate)
	HIV		-	known HIV+
	Substance use		drug use disorder for the past two years	dependence or abuse for the past two years
	Bleeding		-	uncontrolled, e.g. platelets < 50.000 or INR >1.5
MRI exclusions	Microhemorrhage		>4 microhemorrhages	>4 microhemorrhages
	Macrohemorrhage		any macrohemorrhage	any macrohemorrhage (10 mm)
	Siderosis		more than one area of superficial siderosis	any superficial siderosis
	Small vessel disease		severe white matter disease	severe small vessel disease
	Other		any clinically significant finding that would impact safe participation in the study	vasogenic edema, tumefactive lesions, tumor/mass (except small meningioma or cyst), contusion, encephalomalacia, aneurysm, inflammatory amyloid angiopathy

Abbreviations: MCI, mild cognitive impairment; MMSE, Mini-Mental State Examination; PET, positron emission tomography; CSF, cerebrospinal fluid; t-tau, total tau; AChE-I, acetylcholinesterase inhibitors; BMI, body mass index, Aβ, amyloid beta; AD, Alzheimer’s disease; TIA, transient ischemic attack; GDS, geriatric depression scale; HIV, human immunodeficiency virus; TSH, thyroid stimulating hormone; INR, international normalized ratio; MRI, magnetic resonance imaging.

**Box 1. Cases in which specialists may consider anti-amyloid therapies^8^.**

- Clinical diagnosis of MCI or mild dementia due to AD (CDR 0.5 or 1);
- PET or CSF studies positive for amyloid pathology;
- MMSE >20*;
- Patients on cholinesterase inhibitors;
- Partner or family member is available for support; and
- Patient and partner or family member understand the costs, potential benefits, and harms of the treatment.

Abbreviations: AD, Alzheimer’s disease; CDR, Clinical Dementia Rating; MCI, mild cognitive impairment; PET, positron emission tomography; CSF, cerebrospinal fluid; MMSE, Mini-Mental State Examination.Note: *MMSE may vary according to schooling and language impairment, so it should not be used solely for eligibility.

**Box 2. Cases in which specialists should not consider anti-amyloid therapies^8^.**

- Cognitively unimpaired individuals (CDR 0);
- Moderate or advanced dementia (CDR 2 or 3, FAST >4);
- Any medical, neurologic, or psychiatric condition contributing to cognitive decline (e.g., other degenerative dementias, major depression, cerebrovascular disease);
- More than four microhemorrhages <10 mm or one single macrohemorrhage >10 mm on MRI (no longer than 12 months);
- ApoE ε4 homozygosis;
- History of TIA, stroke, or seizures in the past 12 months;
- Any history of immunologic disease or immunosuppressants;
- Anticoagulants, bleeding disorders, platelets <50.000 or INR >1.5; and
- Unstable medical conditions (e.g., cardiac, respiratory, renal frailties).

Abbreviations: ApoE, apolipoprotein-E; CDR, Clinical Dementia Rating; FAST, Functional Assessment Staging Tool; MRI, magnetic resonance imaging; TIA, transient ischemic attack; INR, international normalized ratio.

**Box 3. Minimum resources and reference centers for the safe and effective use of anti-amyloid therapies^8,9^.**

- Dementia specialists (Neurologists, Geriatricians, or Geriatric Psychiatrists) for the diagnosis of MCI or mild dementia due to AD;
- Availability of MRI before and during treatment and trained neuroradiologists for identification and interpretation of cerebrovascular lesions or ARIA;
- Access to amyloid tests (amyloid PET or CSF);
- Access to ApoE genotyping;
- Specialized counseling regarding indications, contraindications, benefits, and risks of treatment;
- Medication infusion resources and a multidisciplinary team trained to recognize and manage infusion reactions; and
- Access to an intensive care unit and experience in the management of neurocritical patients in situations of severe ARIA and standard operating protocols for ARIA.

Abbreviations: AD, Alzheimer’s disease, MCI, mild cognitive impairment; MRI, magnetic resonance imaging; ARIA, amyloid-related neuroimaging abnormalities; ApoE, apolipoprotein E gene; PET, positron emission tomography; CSF, cerebrospinal fluid.

While adherence to strict inclusion and exclusion criteria is mandatory, there is significant debate regarding the use of anti-amyloid therapies outside the studies’ boundaries. This is particularly important concerning how cognitive assessment is performed. For example, some individuals with low educational levels may perform poorly in cognitive screening tests such as the Mini-Mental State Examination (MMSE). Despite well-established MMSE cutoff values for cognitive impairment according to schooling in our populations^21^, such stratification could be insufficient for differentiating mild from moderate dementia. Other potential grey zones for cognitive screening include younger individuals with sporadic AD who are more likely to have atypical clinical syndromes (e.g., logopenic aphasia, dysexecutive/behavioral variant, or posterior cortical atrophy), which can severely influence cognitive test performance^22^. In this sense, we recommend that MMSE (or other screening tests) not be used solely as a proxy for cognitive status and treatment eligibility. It is paramount that functional assessment is undertaken and the activities an individual can perform are within those specified in stages 0.5 or 1.0 of the CDR global score. It is also important to note that MCI patients may perform normally on cognitive screening tests. To differentiate them from cognitively normal individuals (including those with SCD), a battery of neuropsychological tests standardized by age and education should be performed.

### Cautions and adverse events

**Figure 2:**
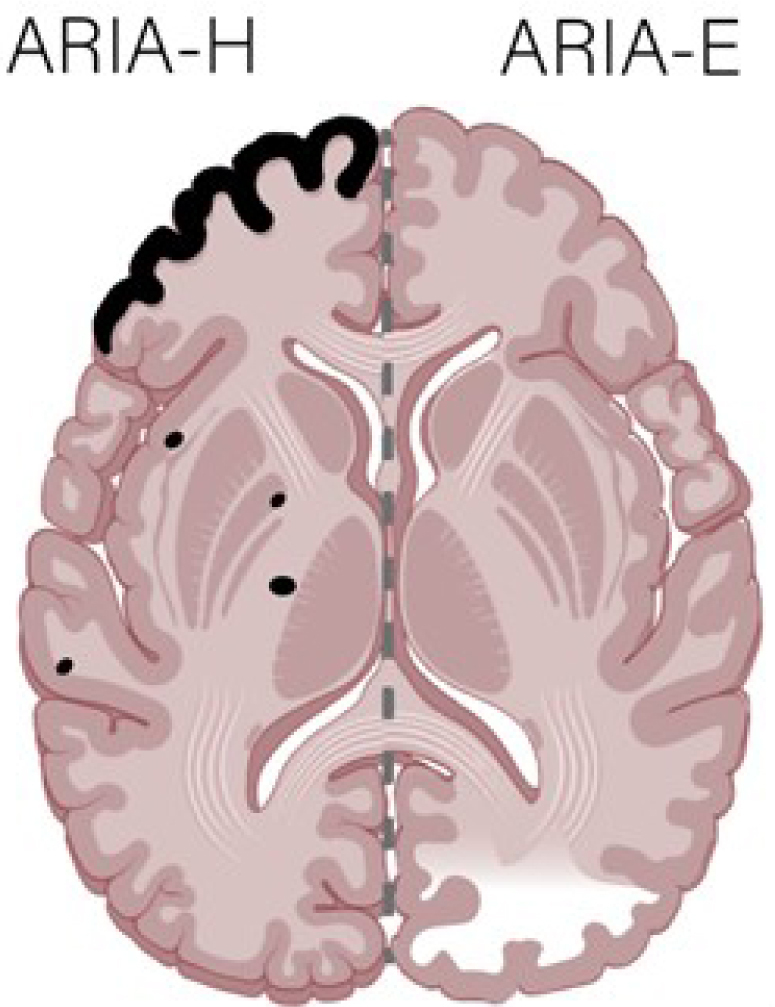
Amyloid-related imaging abnormalities with hemorrhage manifests in magnetic resonance scans as very low-intensity signals detected on gradient echo or susceptibility-weighted imaging magnetic resonance imaging sequences. Amyloid-related imaging abnormalities with edema commonly manifests as hyperintensities on fluid-attenuated inversion recovery or T2-weighted magnetic resonance imaging sequences with no restricted diffusion.

Abbreviations: ARIA-H, amyloid-related imaging abnormalities with hemorrhage; ARIA-E, amyloid-related imaging abnormalities with edema.

Adverse events (AEs) were commonly observed in phase 3 clinical trials with donanemab and lecanemab. The most common AEs were infusion-related reactions (Figure 2), amyloid-related imaging abnormalities with edema (ARIA-E), and ARIA with hemorrhage (ARIA-H). Details of the latter two types of AEs are discussed in the following section.

In the lecanemab phase 3 trial, the most frequent AEs (i.e., affecting >10.0% of the treated patients) were infusion-related reactions (26.4%), headaches (11.1%), and falls (10.4%). Serious AEs occurred in 14.0% of patients in the active treatment arm and 11.3% of those receiving a placebo. AEs that led to discontinuation of the drugs were reported in 6.9% of the lecanemab group *vs.* 2.9% in the placebo group. Six patients (0.7%) died in the lecanemab group and seven (0.8%) in the placebo group, but no deaths were considered related to the drug^4^.

As for donanemab, besides ARIA, the most frequent AEs in the phase 3 study were headaches (14.0%) and falls (13.4%). Infusion-related reactions occurred in 8.7% of donanemab-treated patients. AEs leading to discontinuation of the drug were reported in 8.1% of the donanemab group *vs.* 3.7% in the placebo group. Serious AEs were reported in 17.4% of patients receiving donanemab and in 15.8% of those in the placebo group. Sixteen patients treated with donanemab died during the study, and three deaths (0.4%) were judged related to the treatment. Of these three participants who died, two were apolipoprotein-E (ApoE) ε4 heterozygous carriers, and one was a noncarrier; the causes of all deaths were fatal ARIA-E and ARIA-H. All three had low/medium baseline tau on screening PET scans, and none was prescribed antithrombotic medication. In contrast, ten deaths occurred in the placebo group, with only one (0.1%) considered treatment-related.

More recently, results from the open-label extension (OLE) were published, and there were nine deaths, with four possibly related to study treatment. Of the 24 deaths in the Core study + OLE, three were due to intracerebral hemorrhage (ICH): one placebo in the Core due to ICH, and two lecanemab in OLE with concurrent ICH (one on tissue plasminogen activator and one on anticoagulant therapy). In the Core + OLE, the most common AEs in the lecanemab group (>10%) were infusion-related reactions (24.5%), ARIA with hemosiderin deposits (ARIA-H) microhemorrhages (16.0%), COVID-19 (14.7%), ARIA-E (13.6%), and headache (10.3%)^23^.

After approval of lecanemab, two deaths occurred during clinical treatment in patients homozygous for ApoE ε4 alleles. One case arose after the patient received thrombolysis for stroke^24^. In the other case, the patient developed extensive microhemorrhages and severe cerebral amyloid-related inflammation after three infusions^25^. Previous studies with gantenerumab and bapineuzumab already noticed that ARIA increases with the ApoE ε4 genotype, which strongly argues for caution in these patients^26,27^. Despite not being a formal contraindication to anti-amyloid therapies, we indicate that all eligible patients be tested for ApoE genotype, and the prescription must be based on a careful risk-benefit analysis. Our group advises against using donanemab and lecanemab in individuals with homozygosity for ApoE ε4.

The Appropriate Use Recommendations (AURs) advise that patients be selected for therapy based on criteria similar to those patients who have completed lecanemab clinical trials. This is the population in whom safety and efficacy have been evaluated^8^.

Magnetic resonance imaging (MRI) is fundamental during treatment with anti-amyloid agents. In addition to the usual recommended frequency (see below), it shall be ordered for any time of the treatment when headache, nausea, dizziness, acute confusional state, visual change, seizure, gait change, or any other neurological abnormality judged to be potentially related to the treatment appears.

There are some other important points of caution regarding the AEs of lecanemab. The twice as high drop-out rate in the treatment group due to severe AEs not only risks biasing the efficacy estimates in the endpoint curves but is also a “red flag” itself regarding the risk-benefit balance of lecanemab. Long-term follow-up of all patients, including those who left the trial, is essential for conclusions about the drug’s safety.

Another primary concern is the evidence for brain atrophy in phases 2 and 3 studies with lecanemab, a phenomenon already seen with other antibodies^28^. The explanation is unclear but the amyloid clearance is not very likely the cause, as its accumulation does not induce brain swelling. Some *postmortem* studies in preclinical models and patients indicate that the overall volume of amyloid deposition accounts for less than 1.0% of the neocortex. Segmentation techniques can be applied to quantify brain and cerebrospinal volumes. [^18^F]-Fluorodeoxyglucose positron emission tomography (FDG-PET) imaging data should be combined with MRI to determine brain function and structure following antibody treatments. The lack of rigorous data to rule out brain volume changes due to treatment-related tissue damage is another significant concern for clinicians and patients. A recent meta-analysis emphasizes this issue, indicating that antibody treatments accelerate AD-like changes in brain volume^29^.

Cerebral amyloid angiopathy (CAA) is another concern that arises in the indication and monitoring of patients in the use of these medications. In addition to being related to AD, it also shares pathology with ARIA, which could explain the risk of ARIA associated with the use of anticoagulants in clinical trials.

We must prepare for the eventuality that the real-world effects of lecanemab and donanemab may be much smaller than reported in the trial due to biases, multiple cerebral and systemic comorbidities, and heterogeneity. We believe that AD patients, especially older ones, often have comorbidities that will make them more vulnerable to AE risks and less likely to respond to treatment.

### Amyloid-related neuroimaging abnormalities

MRI is fundamental for monitoring potential AEs for monoclonal antibodies. ARIA is a term used to cover two types of MRI signal alterations: parenchymal edema and sulcal effusion (ARIA-E) and hemorrhage (hemosiderin deposits), including microhemorrhages and leptomeningeal superficial siderosis (ARIA-H). Both alterations are thought to be secondary to the therapeutic effects of monoclonal antibodies, which can increase vascular fragility with the outflow of vascular fluids and red cells. ARIA-E commonly manifests as hyperintensities on fluid-attenuated inversion recovery (FLAIR) or T2-weighted MRI sequences with no restricted diffusion. Increased signal on T2-weighted or FLAIR sequences also occurs in the sulci or leptomeningeal spaces. It may be associated with locoregional mass effect or gyral swelling, and most commonly affects the occipital lobes, followed by the parietal, frontal, and temporal lobes, with rare cerebellar involvement. The MRI findings of ARIA-E are generally transient and resolve upon interruption or discontinuation of therapy, and they may even resolve despite the continuation of treatment^30^. Diseases that may exhibit MRI findings, like those of ARIA-E, include posterior reversible encephalopathy syndrome, evolving subacute ischemia and inflammatory CAA. The availability of a pretreatment T2-weighted or FLAIR sequence is essential to allow later detection of ARIA-E during a clinical trial, especially when the findings of ARIA-E are subtle^30^.

ARIA-H is characterized by hemosiderin, a blood degradation product that manifests as parenchymal microhemorrhages or leptomeningeal superficial siderosis. These MRI findings parallel CAA, including a lobar or peripheral predilection often occurring at the grey matter-white matter junction or cortex. ARIA-H is detected on gradient echo (GRE) or susceptibility-weighted imaging (SWI) MRI sequences as a markedly hypointense signal in the parenchyma or sulci^30^.

The Alzheimer’s Association Research Roundtable Workgroup report suggests a minimal MRI protocol: field strength of at least 1.5 T, acquisition of GRE or T2-weighted and FLAIR sequences, section thickness of 5 mm, and echo time of 20 milliseconds, but it is appropriate to include T1, SWI and diffusion-weighted imaging (DWI)^31^. Considering clinical reports, describing the location and severity of ARIA alterations is essential. It is also helpful to apply objective scales, like, in the case of ARIA-E, the 60-point scoring Barkhof Grand Total Scale or a simplified 3-point scale (and its 5-point variant), which has been used in some clinical trials, like the aducanumab trials^32^. Regarding ARIA-H, it is fundamental to report the number of microbleeds and the presence and location of superficial siderosis. The Microbleed Anatomical Rating Scale is also helpful^33^. Some ARIA severity grading schemes have been proposed with a specific scheme included in the FDA guidance for the clinical use of aducanumab. The FDA guidance defines mild, moderate, and severe ARIA-E and ARIA-H, as shown in Table 4 ^34^.

**Table 4:** Magnetic Resonance Imaging classification of amyloid-related imaging abnormalities^34^.

ARIA type	Radiographic severity		
	Mild	Moderate	Severe
ARIA-E	FLAIR hyperintensity confined to sulcus and/or cortex/subcortex white matter in one location <5 cm	FLAIR hyperintensity 5–10 cm in single greatest dimension, or more than one site of involvement each measuring <10 cm	FLAIR hyperintensity >10 cm with gyral swelling and sulcal effacement. One or more sites may be noted.
ARIA-H microhemorrhages	<4 new incident microhemorrhages	5–9 new incident microhemorrhages	≥10 new incident microhemorrhages
ARIA-H superficial siderosis	One focal area of superficial siderosis	Two focal areas of superficial siderosis	More than two focal areas of superficial siderosis

Abbreviations: FLAIR, fluid-attenuated inversion recovery; ARIA-E, amyloid-related imaging abnormality with edema and effusion; ARIA-H, amyloid-related imaging abnormality with hemosiderin deposits.

ARIA incidence is more frequent in ApoE ε4 carriers. In the aducanumab phase 3 studies ENGAGE and EMERGE^32^, ARIA-E occurred in 42.2% of ApoE ε4 carriers *vs.* 20.3% in noncarriers. Symptoms occurred in 19.2% of cases receiving low doses and 24.4% of subjects receiving high doses. The most common symptoms were headache, dizziness, visual disturbance, nausea, and vomiting. ARIA-H was observed in 6.6% of patients receiving placebo, 16.4% undergoing low-dose aducanumab, and 19.3% taking high-dose aducanumab.

Lecanemab showed a better profile, considering the frequency of ARIA. Based on MRI, ARIA occurred in 12.6% of all participants in the phase 3 CLARITY-AD trial; 2.8% were symptomatic. In the same way as aducanumab, rates of ARIA with symptoms were substantially higher among patients with an ApoE ε4 genotype, especially those homozygous for ApoE ε4. In the recently published OLE data, ARIA-E and ARIA-H were largely radiographically mild-to-moderate. ARIA-E generally occurred within 3–6 months of treatment, was more common in ApoE ε4 carriers (16.8%), and was most common in ApoE ε4 homozygous participants (34.5%)^24^. In the TRAILBLAZER-ALZ 2 clinical trial using donanemab, 36.8% had ARIA E or H, vs. 14.9% using a placebo. Most ARIA-E were mild to moderate and symptomatic in 6.1% of the donanemab group.

Regarding ARIA monitoring, experts recommend performing a pre-treatment MRI within one year before initiating therapies and before the 5th, 7th, 14th, and 26th infusions or in case any symptoms of ARIA occur. Additionally, both ARIA-E and ARIA-H occur early in the treatment course, with ARIA-H often being asymptomatic and detected incidentally on routine MRI surveillance^34^. Investigators suggest awaiting the resolution of ARIA-E and stabilization of ARIA-H before resuming infusions. Permanent discontinuation is advised for macrohemorrhages.

Although ApoE genotyping is not a biomarker of AD, we believe that ApoE testing is mandatory in individuals with clinical indications to use anti-amyloid drugs to estimate the potential risk for ARIA, as detailed in the previous paragraphs.

### Size-effect, cost-effectiveness, and eligibility

The lack of robust clinical significance may raise the question of whether anti-amyloid therapy has a clinical role in the treatment of most AD patients^35^. A subanalysis of groups showed that lecanemab has no benefit (even minimal) in women, black and Asian races, ages under 65 years, and ApoE ε4 homozygosis. The MCI subgroup had less improvement than patients with mild dementia, contrary to expectations that the earlier the treatment the better the response^4^. This was confirmed by a recent meta-analysis of 19 studies with different anti-amyloid agents, which did not find superiority in the clinical effects of MCI compared to mild dementia^36^.

Patients using donanemab or lecanemab had a slower cognitive and functional decline of approximately 30% by 18 months compared to placebo. However, this relative difference equals an absolute 0.45 points difference in the same score (range 0–18). One study estimated that amyloid pathology would be responsible for approximately 30 to 40% of the cognitive decline in patients with AD^37^. Therefore, the gain obtained with these medications is perhaps proportional to what was expected from this therapeutic approach.

A distinction must be made between “statistically significant” and “clinically relevant” effects. Although certain changes may be statistically significant, family members and caregivers may not notice any difference, as no symptomatic effect occurs. A previous study by Liu et al.^38^ suggested that the minimum clinical importance difference (MCID) was 0.98 points in the CDR-SB (within an 18-point range) and 1.26 points in the MMSE (30 points) for patients with MCI and 1.63 points in the CDR-SB and 2.32 in the MMSE for patients with mild dementia. In both studies, the difference between groups was approximately 0.50 points in the CDR-SB, lower than the MCID. Cognitive differences of this magnitude over 18 months have already been observed with donepezil^39^ and multi-nutrient supplements^40,41^. Moreover, positive long-term effects of cholinesterase inhibitors have also been described, such as reduced mortality and reduced risk of developing severe dementia after an average of five years of treatment^42^.

The studies on anti-amyloid drugs suggest a potential benefit, which is a delay in the progression of the disease phase by up to seven months compared to a placebo. It is important to note that in Petersen et al. study in 2005, which focused on the treatment of MCI using vitamin E/donepezil, the group with MCI and ApoE ε4 polymorphism remained stable for a longer period, with this difference being significant for up to three years^39^. In the studies with these monoclonal antibodies (donanemab and lecanemab), the follow-up time was much shorter. We do not have results from patients who no longer receive these medications (after the removal of amyloid) that allow continuation or discontinuation.

With such discrete (and clinically questionable) cognitive benefits and the high annual cost (the cost of medication alone is greater than US$25,000 per year for lecanemab and US$32,000 for donanemab), several studies have shown that these medications are not cost-effective^43,44,45^. One recent article suggests that the annual price of lecanemab should be up to US$5.100/year to be cost-effective^46^. This discussion is fundamental when evaluating pricing in Brazil. Our group emphasizes the need to conduct cost-effectiveness studies in LMIC since they were not included in most clinical trials.

A final aspect that deserves attention is the eligibility of patients in real-life settings to receive treatment with anti-amyloid drugs. In a recent study, the authors applied the inclusion and exclusion criteria of aducanumab and lecanemab trials to 237 patients with MCI or mild AD from a population-based study. They found that only 8.0% of subjects were eligible for lecanemab, while 5.1% were eligible for aducanumab treatment^47^.

### What are the reasons for the repeated failures of randomized clinical trials with anti-amyloid drugs for ad treatment?

A swift analysis of the available trials with anti-amyloid immune compounds prompts that the clinical benefits fell short of expectations despite accomplishing the expected biological outcome (i.e., amyloid clearance from the brain). Methodological shortcomings were proposed to explain the limited (or lacking) efficacy of several well-conducted, large-scale, randomized clinical trials with these compounds. Criticisms range from identifying flaws in the experimental design to questioning core neurobiological assumptions within the disease model^48^. On the bottom line, some authors suggest that the “amyloid cascade hypothesis” may be wrong or insufficient to fully explain the pathogenesis of sporadic AD. Addressing these concerns will help to improve the success rate in AD drug development, particularly in passive immunotherapy trials, which has been the most successful disease-modifying approach^49,50^.

Developing new treatments for AD may require multiple amendments to the current state of the art. These should start from optimizing the recruitment processes, aiming at a more accurate and specific selection of eligible patients (i.e., those with a clinical-biological profile more likely to respond to anti-amyloid drugs) and having a better comprehension of the dynamics of treatment outcomes so that clinical trial designs can be adjusted to encompass more realistic endpoints^51^. In the forthcoming years, the AD drug development pipeline will incorporate a diversification of molecular targets and combined therapies and non-pharmacological treatments to simultaneously intervene in multiple disease mechanisms that pertain to the complex AD neurobiology. The timing of the intervention will be crucial to avoid recapitulating the failure of recent trials with anti-amyloid compounds. The complexity of the disease — including, but not limited to, amyloid accumulation — highlights the necessity of multi-target and multi-modal interventions, reducing the gap between experimental and clinically-targeted interventions with disease-modifying strategies. Finally, it is essential to emphasize that co-pathologies (e.g., vascular and other neurodegenerative proteinopathies) are very common among older adults with MCI or dementia and contribute significantly to the clinical manifestations^52,53^.

### Populational diversity and clinical trials in low- or middle-income countries (particularly Brazil)

An additional component worth noting when analyzing the clinical effect of anti-amyloid trials is the need for more representation of populations outside North America and Europe. A study in 2013 showed that among 715 worldwide AD clinical trials, only 34 were performed in South America and eight in Brazil^54^. Similarly, a 2020 study showed that only 6.0% of dementia clinical trials occurred in Latin America, primarily in Argentina, Brazil, Chile, Colombia, and Mexico^55^. A recent systematic review of the distribution of AD and related dementias (ADRD) clinical trials showed that less than 3.0% of trials were conducted in Latin America and Africa, mainly phases 2/3 and 3, while 52.8% of all phase 1 were in North America and 29.9% in Europe^56,57^. These figures highlight the inequity in ADRD clinical trials in LMICs.

The main barriers to conducting clinical trials in LMIC, like Brazil, are operational (unsupportive administrative system, lack of skilled administrative personnel); regulatory (delay in approval decisions, complex and inefficient regulatory system); resources (shortage of funding, infrastructure, research materials); population (lack of awareness and trust in science); and individual (absence of motivation, need for leadership, lack of interest by policymakers)^57^.

As individuals living in LMICs are often underrepresented in dementia clinical trials, their relevance is limited due to factors such as ethnicity, socioeconomic diversity, general health, comorbidities, and nutrition. For example, educational level and cognitive reserve could affect the rate of progression. Variations in healthcare access are also essential to consider, including access to biomarkers, MRI, and the availability of a precise diagnosis. Additionally, the pharmacokinetic and pharmacodynamic aspects are imperative to understanding a drug’s effect, as they may differ among ethnic backgrounds. It is known that compound sensitivity may change due to several factors related to ethnic background, such as non-linear pharmacokinetics, narrow therapeutic dose range, metabolism by enzymes known to show genetic polymorphism, and finally, low bioavailability, and more susceptibility to dietary absorption effects. As a crucial difference from high-income countries, the Brazilian population has a different ApoE ε4 allele frequency and may be at higher risk for ARIA-E and ARIA-H^58,59^.

Recent developments in AD disease-modifying interventions have opened up a new landscape in AD diagnosis, care, and treatment. Greater collaboration among primary care clinicians and specialists will be required, with infusion centers being adequate places to offer therapy^7^.

The use of these new therapies in Brazil is challenged by factors such as delay in MCI and dementia diagnosis, a higher burden of vascular pathologies^53^, costs, and a shortage of dementia specialists. A particularly challenging scenario for this discussion is the implementation of anti-amyloid therapies in Brazil’s Unified Health System (SUS, *Sistema Único de Saúde*). The SUS provides free healthcare to all Brazilian citizens and has been instrumental in improving access to healthcare services for many people, particularly those living in poverty. Through prevention, immunization, research, and education initiatives, the system has reached significant milestones in the past decades, such as:

- The reduction of infant mortality rates;
- Improvements in infectious disease control, such as human immunodeficiency virus/ acquired immunodeficiency syndrome (HIV/AIDS), malaria, and tuberculosis^60^; and
- Approval of thrombectomy for stroke (after a proper multicentric study^61^).

On the other hand, Brazil still lags behind many other countries in terms of population coverage, quality metrics, and overall and efficient spending.

As discussed by Mattke et al.^56^, a significant obstacle is the scarce accessibility of dementia specialists. The growth of the older population means that the waiting list for specialist appointments will continue to increase. The adequacy of the necessary infrastructure for both diagnosis (availability of biomarkers, MRI, ApoE genotyping) and treatment (availability of infusion centers and the need for frequent MRI) to attend to the entire potentially eligible population is a challenge for both universal public health systems and supplementary health systems (health insurance companies). Even with theoretical accessibility to biomarker testing and treatment, the estimated waiting time for treatment could reach two years on average, with substantial differences between the public and private sectors, as capacity growth is insufficient to keep up with increasing demand.

Given the high costs of medication, infrastructure needs, and the high prevalence of AD in a country with an underfunded universal health system, cost-effectiveness studies are necessary to approve anti-amyloid monoclonal antibodies. For example, despite positive results in other countries, implementing thrombectomy in the Brazilian public health system followed a similar process, with the government mandating a clinical trial to determine safety and efficacy before approval^61^. Therefore, we propose testing anti-amyloid drugs in selected reference centers in Brazil to validate efficacy, safety, and cost-effectiveness before potential approval by public and private health systems. Therefore, we propose that anti-amyloid drugs undergo testing in selected reference centers in Brazil before potential approval by public and private health systems to validate the treatment’s efficacy, safety, and cost-effectiveness.

## CONCLUSIONS AND RECOMMENDATIONS

- To date, only donanemab and lecanemab have been approved by the FDA as disease-modifying therapies for AD treatment. However, the EMA has recommended the refusal of marketing authorization for lecanemab. Approval is still pending in other countries like Japan, China, and Brazil.
- Proven amyloid pathology is pivotal to prescribing anti-amyloid drugs. Amyloid pathology may be identified with PET or lumbar puncture with CSF analysis;
- ApoE testing must be performed in individuals with clinical indications to use anti-amyloid drugs to estimate the potential risk for ARIA. Our group advises against using donanemab and lecanemab in individuals with homozygosity for ApoE ε4.
- The most fundamental principle is that potential candidates for anti-amyloid therapies should be in the early, symptomatic stages of AD, namely MCI or mild dementia.
- These medications should not be prescribed to cognitively unimpaired individuals (e.g., asymptomatic or those with SCD), nor to patients with AD in moderate or severe stages.
- Concerned with inappropriate, widespread use of donanemab or lecanemab, we strongly suggest strictly following the inclusion and exclusion criteria of the trials for each drug.
- Reference centers for the safe and effective use of anti-amyloid therapies must be prepared with at least minimum resources, including dementia specialists, patient counseling, neuroimaging monitoring, infusion, and AEs protocols.
- AEs were commonly observed in phase 3 clinical trials with donanemab and lecanemab. The most common were infusion-related reactions, ARIA-E and ARIA-H.
- Regarding ARIA monitoring, a pre-treatment MRI should be obtained within one year before initiating therapies and before the 5th, 7th, 14th, and 26th infusions of lecanemab. As for donanemab, MRI is warranted before the 4th, 12nd, and 24th weeks of treatment (or whenever symptoms of ARIA occur).
- The lack of robust clinical significance raises the question of whether anti-amyloid therapy has a clinical role in the treatment of most AD patients. With modest cognitive and functional benefits and high annual costs, several studies suggest that these medications are not cost-effective.
- As individuals living in LMICs are often underrepresented in dementia clinical trials, factors such as ethnicity, socioeconomic diversity, general health, comorbidities, and nutrition may affect equity for anti-amyloid treatments and differences in clinical effectiveness.
- We suggest that anti-amyloid drugs be tested in select reference centers in Brazil before eventual approval of public and private health systems to validate treatments’ efficacy and cost-effectiveness.

## SUPPLEMENTARY MATERIAL

Use of anti-amyloid therapies for Alzheimer’s disease in Brazil: a position paper from the Scientific Department of Cognitive Neurology and Aging of the Brazilian Academy of Neurology, available at: https://www.demneuropsy.com.br/wp-content/uploads/2024/10/DN-2024.C002-PT.pdf

## References

[1] Self WK, Holtzman DM (2023). Emerging diagnostics and therapeutics for Alzheimer disease. Nat Med.

[2] Rabinovici GD, La Joie R (2023). Amyloid-targeting monoclonal antibodies for Alzheimer disease. JAMA.

[3] Biogen Biogen to realign resources for Alzheimer’s disease franchise [Internet].

[4] van Dyck CH, Swanson CJ, Aisen P, Bateman RJ, Chen C, Gee M (2023). Lecanemab in Early Alzheimer’s Disease. N Engl J Med.

[5] European Medicines Agency (2024). Leqembi [Internet].

[6] Sims JR, Zimmer JA, Evans CD, Lu M, Ardayfio P, Sparks J (2023). Donanemab in early symptomatic Alzheimer disease: the TRAILBLAZER-ALZ 2 randomized clinical trial. JAMA.

[7] Brucki SMD, César-Freitas KG, Spera RR, Borges CR, Smid J (2022). Are we ready to use anti-amyloid therapy in Alzheimer’s disease?. Arq Neuropsiquiatr.

[8] Cummings J, Apostolova L, Rabinovici GD, Atri A, Aisen P, Greenberg S (2023). Lecanemab: appropriate use recommendations. J Prev Alzheimers Dis.

[9] Cummings J, Salloway S (2022). Aducanumab: appropriate use recommendations. Alzheimers Dement.

[10] Jack CR, Bennett DA, Blennow K, Carrillo MC, Dunn B, Haeberlein SB (2018). NIA-AA research framework: toward a biological definition of Alzheimer’s disease. Alzheimers Dement.

[11] Hansson O, Seibyl J, Stomrud E, Zetterberg H, Trojanowski JQ, Bittner T (2018). CSF biomarkers of Alzheimer’s disease concord with amyloid-β PET and predict clinical progression: A study of fully automated immunoassays in BioFINDER and ADNI cohorts. Alzheimers Dement.

[12] Minoshima S, Drzezga AE, Barthel H, Bohnen N, Djekidel M, Lewis DH (2016). SNMMI Procedure Standard/EANM Practice Guideline for Amyloid PET imaging of the brain 1.0. J Nucl Med.

[13] Coutinho AM, Busatto GF, Porto FHG, Faria DP, Ono CR, Garcez AT (2020). Brain PET amyloid and neurodegeneration biomarkers in the context of the 2018 NIA-AA research framework: an individual approach exploring clinical-biomarker mismatches and sociodemographic parameters. Eur J Nucl Med Mol Imaging.

[14] La Joie R, Ayakta N, Seeley WW, Borys E, Boxer AL, DeCarli C (2019). Multisite study of the relationships between antemortem [11C]PIB-PET Centiloid values and postmortem measures of Alzheimer’s disease neuropathology. Alzheimers Dement.

[15] Klunk WE, Koeppe RA, Price JC, Benzinger TL, Devous MD, Jagust WJ (2015). The centiloid project: standardizing quantitative amyloid plaque estimation by PET. Alzheimers Dement.

[16] Hazan J, Wing M, Liu KY, Reeves S, Howard R (2023). Clinical utility of cerebrospinal fluid biomarkers in the evaluation of cognitive impairment: a systematic review and meta-analysis. J Neurol Neurosurg Psychiatry.

[17] Hansson O, Batrla R, Brix B, Carrillo MC, Corradini V, Edelmayer RM (2021). The Alzheimer’s Association international guidelines for handling of cerebrospinal fluid for routine clinical measurements of amyloid β and tau. Alzheimers Dement.

[18] Hansson O, Edelmayer RM, Boxer AL, Carrillo MC, Mielke MM, Rabinovici GD (2022). The Alzheimer’s Association appropriate use recommendations for blood biomarkers in Alzheimer’s disease. Alzheimers Dement.

[19] Mattsson-Carlgren N, Collij LE, Stomrud E, Binette AP, Ossenkoppele R, Smith R (2024). Plasma biomarker strategy for selecting patients with Alzheimer disease for antiamyloid immunotherapies. JAMA Neurol.

[20] Hampel H, Hu Y, Cummings J, Mattke S, Iwatsubo T, Nakamura A (2023). Blood-based biomarkers for Alzheimer’s disease: current state and future use in a transformed global healthcare landscape. Neuron.

[21] Wolk DA, Sadowsky C, Safirstein B, Rinne JO, Duara R, Perry R (2018). Use of flutemetamol F 18-labeled positron emission tomography and other biomarkers to assess risk of clinical progression in patients with amnestic mild cognitive impairment. JAMA Neurol.

[22] Brucki SMD, Nitrini R, Caramelli P, Bertolucci PHF, Okamoto IH (2003). Suggestions for utilization of the mini-mental state examination in Brazil. Arq Neuropsiquiatr.

[23] Ramanan VK, Armstrong MJ, Choudhury P, Coerver KA, Hamilton RH, Klein BC (2023). Antiamyloid monoclonal antibody therapy for alzheimer disease: emerging issues in neurology. Neurology.

[24] Honig LS, Sabbagh MN, van Dyck CH, Sperling RA, Hersch S, Matta A (2024). Updated safety results from phase 3 lecanemab study in early Alzheimer’s disease. Alzheimers Res Ther.

[25] Reish NJ, Jamshidi P, Stamm B, Flanagan ME, Sugg E, Tang M (2023). Multiple cerebral hemorrhages in a patient receiving lecanemab and treated with t-PA for stroke. N Engl J Med.

[26] Solopova E, Romero-Fernandez W, Harmsen H, Ventura-Antunes L, Wang E, Shostak A (2023). Fatal iatrogenic cerebral β-amyloid-related arteritis in a woman treated with lecanemab for Alzheimer’s disease. Nat Commun.

[27] Sperling R, Salloway S, Brooks DJ, Tampieri D, Barakos J, Fox NC (2012). Amyloid-related imaging abnormalities in patients with Alzheimer’s disease patients treated with bapineuzumab: a retrospective analysis. Lancet Neurol.

[28] Sato K, Niimi Y, Ihara R, Suzuki K, Iwata A, Iwatsubo T (2024). APOE-ε4 allele[s]-associated adverse events reported from placebo arm in clinical trials for Alzheimer’s disease: implications for anti-amyloid beta therapy. Front Dement.

[29] Alves F, Kalinowski P, Ayton S (2023). Accelerated brain volume loss caused by anti-β-amyloid drugs: a systematic review and meta-analysis. Neurology.

[30] Roytman M, Mashriqi F, Al-Tawil K, Schulz PE, Zaharchuk G, Benzinger TLS (2023). Amyloid-related imaging abnormalities: an update. AJR Am J Roentgenol.

[31] Sperling RA, Jack CR, Black SE, Frosch MP, Greenberg SM, Hyman BT (2011). Amyloid-related imaging abnormalities in amyloid-modifying therapeutic trials: recommendations from the Alzheimer’s Association Research Roundtable Workgroup. Alzheimers Dement.

[32] Budd Haeberlein S, Aisen PS, Barkhof F, Chalkias S, Chen T, Cohen S (2022). Two randomized phase 3 studies of aducanumab in early Alzheimer’s disease. J Prev Alzheimers Dis.

[33] Gregoire SM, Chaudhary UJ, Brown MM, Yousry TA, Kallis C, Jäger HR (2009). The Microbleed Anatomical Rating Scale (MARS): reliability of a tool to map brain microbleeds. Neurology.

[34] Filippi M, Cecchetti G, Spinelli EG, Vezzulli P, Falini A, Agosta F (2022). amyloid-related imaging abnormalities and β-amyloid-targeting antibodies: a systematic review. JAMA Neurol.

[35] Rubin R (2023). Who should-and can-get lecanemab, the new Alzheimer disease drug?. JAMA.

[36] Dantas JM, Mutarelli A, Navalha DDP, Dagostin CS, Romeiro PHCL, Felix N (2024). Efficacy of anti-amyloid-ß monoclonal antibody therapy in early Alzheimer’s disease: a systematic review and meta-analysis. Neurol Sci.

[37] Zhang W, Wang HF, Kuo K, Wang L, Li Y, Yu J (2023). Contribution of Alzheimer’s disease pathology to biological and clinical progression: a longitudinal study across two cohorts. Alzheimers Dement.

[38] Liu KY, Schneider LS, Howard R (2021). The need to show minimum clinically important differences in Alzheimer’s disease trials. Lancet Psychiatry.

[39] Petersen RC, Thomas RG, Grundman M, Bennett D, Doody R, Ferris S (2005). Vitamin E and donepezil for the treatment of mild cognitive impairment. N Engl J Med.

[40] Soininen H, Solomon A, Visser PJ, Hendrix SB, Blennow K, Kivipelto M (2017). 24-month intervention with a specific multinutrient in people with prodromal Alzheimer’s disease (LipiDiDiet): a randomised, double-blind, controlled trial. Lancet Neurol.

[41] Soininen H, Solomon A, Visser PJ, Hendrix SB, Blennow K, Kivipelto M (2021). 36-month LipiDiDiet multinutrient clinical trial in prodromal Alzheimer’s disease. Alzheimers Dement.

[42] Xu H, Garcia-Ptacek S, Jönsson L, Wimo A, Nordström P, Eriksdotter M (2021). Long-term effects of cholinesterase inhibitors on cognitive decline and mortality. Neurology.

[43] Ross EL, Weinberg MS, Arnold SE (2022). Cost-effectiveness of aducanumab and donanemab for early Alzheimer disease in the US. JAMA Neurol.

[44] Sinha P, Barocas JA (2022). Cost-effectiveness of aducanumab to prevent Alzheimer’s disease progression at current list price. Alzheimers Dement (N Y).

[45] Cliff ERS, Kelkar AH (2022). Cost-effectiveness of aducanumab and donanemab for early Alzheimer disease-estimating the true value. JAMA Neurol.

[46] Nguyen HV, Mital S, Knopman DS, Alexander GC (2024). Cost-effectiveness of lecanemab for individuals with early-stage Alzheimer disease. Neurology.

[47] Pittock RR, Aakre JA, Castillo AM, Ramanan VK, Kremers WK, Jack CR (2023). Eligibility for anti-amyloid treatment in a population-based study of cognitive aging. Neurology.

[48] Loureiro JC, Pais MV, Stella F, Radanovic M, Teixeira AL, Forlenza OV (2020). Passive antiamyloid immunotherapy for Alzheimer’s disease. Curr Opin Psychiatry.

[49] Cummings JL, Morstorf T, Zhong K (2014). Alzheimer’s disease drug-development pipeline: few candidates, frequent failures. Alzheimers Res Ther.

[50] Kim CK, Lee YR, Ong L, Gold M, Kalali A, Sarkar J (2022). Alzheimer’s disease: key insights from two decades of clinical trial failures. J Alzheimers Dis.

[51] Schneider LS (2023). What the gantenerumab trials teach us about Alzheimer’s treatment. N Engl J Med.

[52] Karanth S, Nelson PT, Katsumata Y, Kryscio RJ, Schmitt FA, Fardo DW (2020). Prevalence and clinical phenotype of quadruple misfolded proteins in older adults. JAMA Neurol.

[53] Suemoto CK, Ferretti-Rebustini REL, Rodriguez RD, Leite REP, Soterio L, Brucki SMD (2017). Neuropathological diagnoses and clinical correlates in older adults in Brazil: a cross-sectional study. PLoS Med.

[54] Allegri RF, Bagnati P, Brucki S, Nitrini R, Bairu M, Weiner MW (2014). Global clinical trials for Alzheimer’s disease.

[55] Parra MA, Baez S, Sedeño L, Gonzalez Campo C, Santamaría-García H, Aprahamian I (2021). Dementia in Latin America: paving the way toward a regional action plan. Alzheimers Dement.

[56] Mattke S, Santos OC, Hanson M, Mateus EF, Reis JP, Souza LC (2023). Preparedness of the Brazilian health-care system to provide access to a disease-modifying Alzheimer’s disease treatment. Alzheimers Dement.

[57] Llibre-Guerra JJ, Heavener A, Brucki SMD, Marante JPD, Pintado-Caipa M, Chen Y (2023). A call for clinical trial globalization in Alzheimer’s disease and related dementia. Alzheimers Dement.

[58] Belloy ME, Napolioni V, Greicius MD (2019). A quarter century of APOE and Alzheimer’s disease: progress to date and the path forward. Neuron.

[59] Bahia VS, Kok F, Marie SN, Shinjo SO, Caramelli P, Nitrini R (2008). Polymorphisms of APOE and LRP genes in Brazilian individuals with Alzheimer disease. Alzheimer Dis Assoc Disord.

[60] Roman A (2023). A closer look into Brazil’s healthcare system: what can we learn?. Cureus.

[61] Martins SO, Mont’Alverne F, Rebello LC, Abud DG, Silva GS, Lima FO (2020). Thrombectomy for stroke in the public health care system of Brazil. N Engl J Med.

